# ^1^H NMR metabolomic study of auxotrophic starvation in yeast using Multivariate Curve Resolution-Alternating Least Squares for Pathway Analysis

**DOI:** 10.1038/srep30982

**Published:** 2016-08-03

**Authors:** Francesc Puig-Castellví, Ignacio Alfonso, Benjamin Piña, Romà Tauler

**Affiliations:** 1 Department of Environmental Chemistry, Institute of Environmental Assessment and Water Research, (IDAEA-CSIC), Jordi Girona 18-26, 08034 Barcelona, Catalonia, Spain; 2 Department of Biological Chemistry and Molecular Modelling, Institute of Advanced Chemistry of Catalonia (IQAC-CSIC), Jordi Girona 18-26, 08034 Barcelona, Catalonia, Spain

## Abstract

Disruption of specific metabolic pathways constitutes the mode of action of many known toxicants and it is responsible for the adverse phenotypes associated to human genetic defects. Conversely, many industrial applications rely on metabolic alterations of diverse microorganisms, whereas many therapeutic drugs aim to selectively disrupt pathogens’ metabolism. In this work we analyzed metabolic changes induced by auxotrophic starvation conditions in yeast in a non-targeted approach, using one-dimensional proton Nuclear Magnetic Resonance spectroscopy (^1^H NMR) and chemometric analyses. Analysis of the raw spectral datasets showed specific changes linked to the different stages during unrestricted yeast growth, as well as specific changes linked to each of the four tested starvation conditions (L-methionine, L-histidine, L-leucine and uracil). Analysis of changes in concentrations of more than 40 metabolites by Multivariate Curve Resolution – Alternating Least Squares (MCR-ALS) showed the normal progression of key metabolites during lag, exponential and stationary unrestricted growth phases, while reflecting the metabolic blockage induced by the starvation conditions. In this case, different metabolic intermediates accumulated over time, allowing identification of the different metabolic pathways specifically affected by each gene disruption. This synergy between NMR metabolomics and molecular biology may have clear implications for both genetic diagnostics and drug development.

Metabolomics aims to identify the specific cellular processes undergoing in biological organisms by the identification and quantitation of dozens to thousands metabolites with high-throughput techniques, by using a non-aprioristic approach^1^. Metabolomic analyses have been performed in many organisms, including human and mammalian tissues^2^,^3^, different animal species, both vertebrates^4^ and invertebrates^5^, plants^6^, and microorganisms, both Eukaryotes (yeasts^7^, protists^8^) and Prokaryotes (bacteria^9^, archea^10^).

Among the eukaryotic microorganisms, the yeast *Saccharomyces cerevisiae* is widely used in many biological fields, such as biotechnology^11^ or food industry^12^, and it constitutes an excellent model organism for metabolomics^13^ and other “omic” approaches^14^. We present here an NMR analysis of the metabolome variations induced by auxotrophic starvation in yeast, which occurs when a strain lacking specific genes (in this case, *HIS3, LEU2, MET15* and *URA3*, also called genetic markers) is confronted with a medium devoid of one or more of the essential metabolites it can no longer synthesize (L-histidine, L-leucine, L-methionine and/or uracil, respectively). Reports of metabolic disruption in yeast have previously focused on the triggered proteins^15^ or on the transcriptomic^16^ evidences. The general consensus is that starvation is controlled through the RAS/protein kinase A (PKA) and TOR pathways^17^ that mediate the transcriptional, translational, and metabolic state of the cell. However, most studies analyzed the effects of the depletion in a requiring nutrient, like carbon, nitrogen, phosphate or sulphur sources^14^,^18^. Our analysis is focused on the final downstream product of the whole biological system (the metabolites), which are closer to the final phenotype than RNA or proteins^19^. In addition, the study of different auxotrophic starvations allows a more detailed and specific analysis of the metabolic changes induced by characterized disruptions of the endogenous metabolic pathways.

Conventional technologies for metabolomic analyses include Nuclear Magnetic Resonance (NMR)^20^ or hyphenated techniques of Liquid Chromatography^13^, Gas Chromatography^9^ or Capillary Electrophoresis^7^ coupled to Mass Spectrometry (MS). Despite the fact that NMR allows to identify a quite lower number of metabolites than the maximal capacity of MS^21^, the set of identified metabolites with NMR comprises a broad range of molecules, resulting advantageous for explaining the changes occurring in the cell, whereas for MS a preliminary selection of the peaks (using targeted or non-targeted approaches) has to be performed and the final metabolic overview might result biased.

A previous study has proven the capacity of ^1^H NMR to identify metabolic variations related to distinct genetic backgrounds^22^. The most relevant limitation of NMR, in comparison to MS-derived techniques, is that most of the detectable metabolites are related to primary metabolism, whereas the less abundant ones may be left unobserved due to the relatively low sensitivity of the technique. However, if the studied condition is drastic enough, target metabolites might be raised to detectable concentrations. In addition, and because of the intrinsic properties of NMR, known resonances can be used for quantifying the corresponding metabolites, whereas chemical structures can be deduced from the not-yet-assigned resonances. In this article we applied ^1^H NMR to the study of local disruptions of metabolic pathways, an approach that can be extrapolated to determine the specific triggers for other locally disrupted processes.

By combining the information obtained from the previous metabolic profiling with advanced data analyses, distinct metabolic profiles can be obtained rather than only specific metabolic markers for each studied condition. Here we took advantage of the chemometric approach MCR-ALS^23^ (Multivariate Curve Resolution Alternating Least Squares) to capture the synergistic metabolic patterns for every studied condition. In fluxomics, MCR-ALS has been applied to study reaction contributions of known pathways^24^. In metabolomics, MCR-ALS has been already applied to resolve most of the metabolites from complex HPLC-MS samples^13^, as well as to identify directly the biological profiles from a raw ^1^H NMR dataset^25^. While the use of raw data may allow a more holistic approach to detect unknown changes on the metabolome profile, the use of the concentration areas estimates obtained from peak integration, as it is presented here, can simplify the interpretation of the results.

## Results

### Kinetic analysis of raw ^1^H NMR data

**Figure 1:**
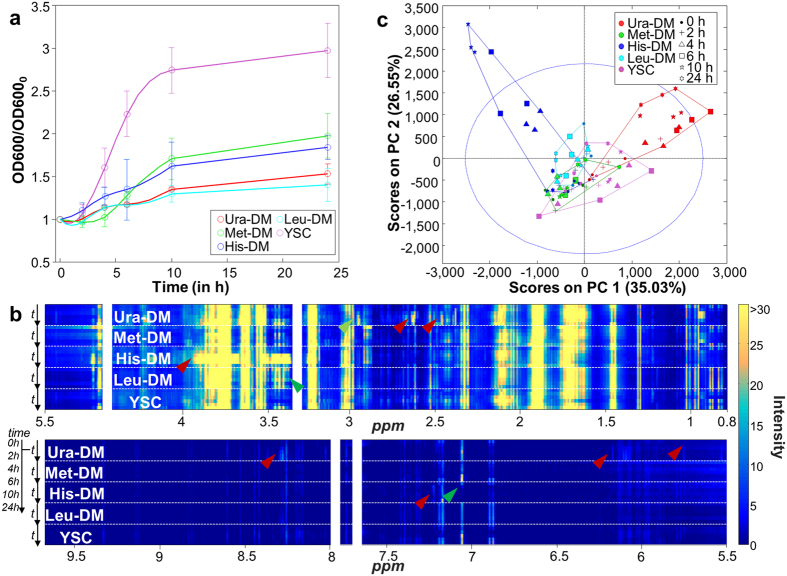
Yeast growth. (**a**) Graphic representation of cell growth at the different media (normalized for the initial OD_600_). (**b**) Heatmap representation of the ^1^H NMR spectra for the five different time-courses. Each row corresponds to the average of 3 spectra. Within each time-course studied, data are presented row-wise following an increasing time order. The red and green arrows point to peak signals of some metabolites accumulated (increasing intensity) or consumed (decreasing) over time. (**c**) PCA scores projection of the mean-centered spectral dataset on PC1 and PC2 subspace. DM, Drop-out Media; YSC, Yeast Nitrogen Base Synthetic Complete medium.

Lack of each one of different auxotrophic markers resulted in a reduction of cell growth, probably reflecting the depletion of the respective internal pools (Fig. 1a). In this context, the drop-out medium lacking L-leucine (Leu-DM) appeared as the most restrictive condition, as it reached only 140% of the original OD after 24 h of culture (Fig. 1a, cyan line). Conversely, the medium lacking L-methionine (Met-DM) allowed growth to reach almost 200% of the original OD after 24 h (Fig. 1a, green line). In contrast, growing in non-restrictive conditions (YSC medium) allowed a vigorous growth for more than 10 hours, reaching more than 250% of the original OD before entering in the stationary phase, likely imposed by the consumption of the available fermentable carbon source (Fig. 1a, purple line).

Metabolic changes during the 24 h-incubation periods in all five conditions were first analyzed by a global overview of the ^1^H NMR spectral dataset (Fig. 1b). The data show examples of proton resonances increasing (red arrows) or decreasing (green arrows) during yeast growth that are characteristic for each physiological condition tested.

^1^H NMR spectra were also analyzed by Principal Component Analysis (PCA). Two components explained 61.6% of the total variability, most of it associated to metabolomic changes in yeast cultured in His-DM and Ura-DM (Fig. 1c, blue and red symbols), but only for samples taken at least after four hours of incubation. We interpret this as indicating accumulation of specific metabolites over time for these two particular auxotrophic starvation conditions. In contrast, a similar analysis performed only for samples from the YSC medium showed a quasi-cyclic variation of the yeast metabolome, in which samples taken at the late stationary phase (24 h) were more similar to the initial inoculum (0 h) than samples taken during the exponentially growing (log) phase (2–10 h) (Supplementary Fig. S1, see also purple symbols in Fig. 1c).

**Figure 2:**
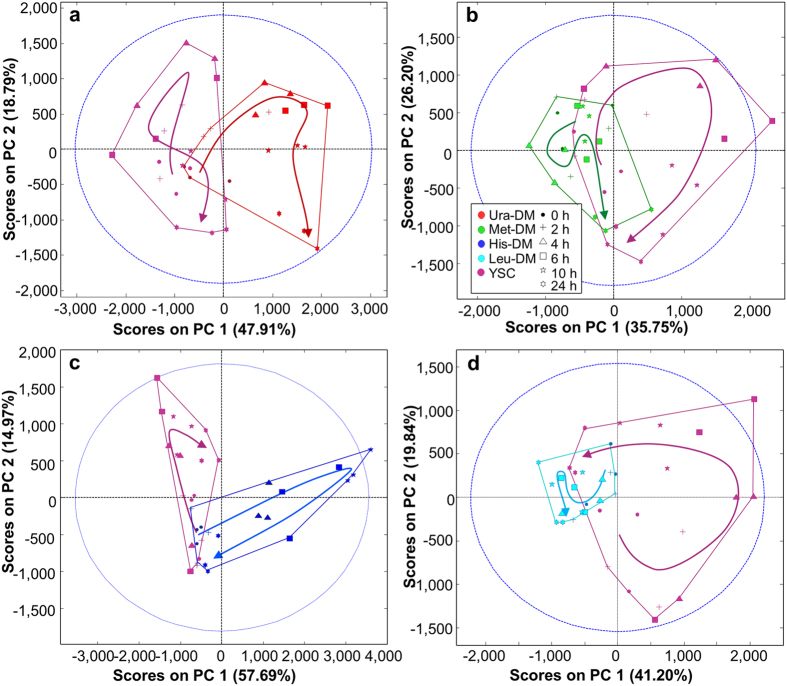
PCA analyses of the metabolomic variance for each starved condition. PC1 and PC2 scores projection of control samples and of samples from yeast cultured in (**a**) Ura-DM, (**b**) Met-DM, (**c**) His-DM, and in (**d**) Leu-DM.

The temporal changes of yeast metabolome under the different starving conditions can be better observed on the four score plots in Fig. 2, corresponding to PCA analyses combining data from each starving condition and the control samples. These plots show the relatively small temporal variability of Leu-DM or Met-DM samples, particularly when compared to their Ura-DM or His-DM counterparts.

Permutation tests using the ASCA method (see *Material and Methods* section), confirmed a significant interaction (p ≤ 0.018) between *time* and *yeast medium*, either for the complete dataset or when using partial datasets, including any of the drop-out media versus control samples, confirming the temporal metabolome variability associated to the different growth conditions (Supplementary Fig. S2).

### Metabolite assignment and quantification

**Figure 3:**
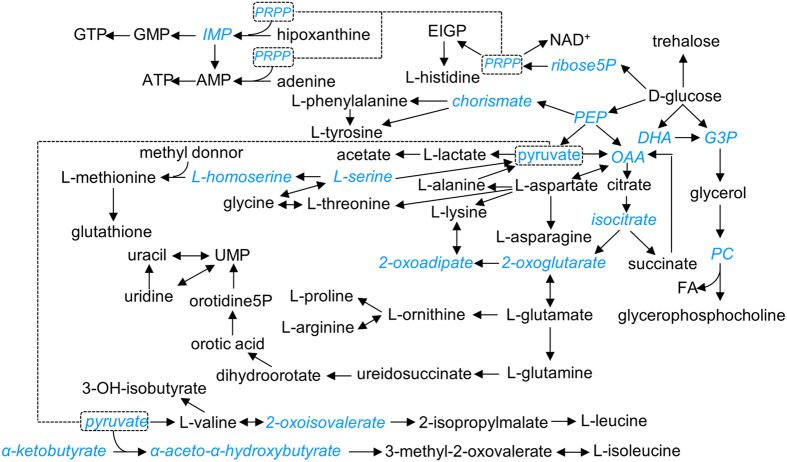
Pathway diagram representing the main interconnections for the assigned metabolites. Assigned metabolites are written in black, whereas non-assigned ones are written in blue italic letters. Solid arrows connect metabolites from a same metabolic pathway, showed here in a simplified way. Dashed arrows connect different pathways sharing a same metabolite.

An exhaustive assignment process (see *Material and Methods* and Supplementary Methods) for the resonances from the NMR spectra allowed for the identification and determination of a total of 47 metabolites. In addition, concentrations from three additional peak resonances were estimated but not unequivocally assigned. Tentative candidates for these three metabolites were deduced from their respective chemical shifts (2.10 ppm, 8.03 ppm and 8.37 ppm) and multiplicities (singlet for all the cases). We propose that the first signal corresponds to a methyl donor of structure R-S-CH_3_, whereas the remaining two correspond to modified purine rings with only one detectable proton, such as isoguanine or xanthine. A table containing the list of metabolites with the identified features in the spectrum is presented in Supplementary Table S1, whereas relative concentration plots are presented in Supplementary Fig. S3. A biological overview of the main interconnections for these metabolites in yeast can be found in Fig. 3.

**Figure 4:**
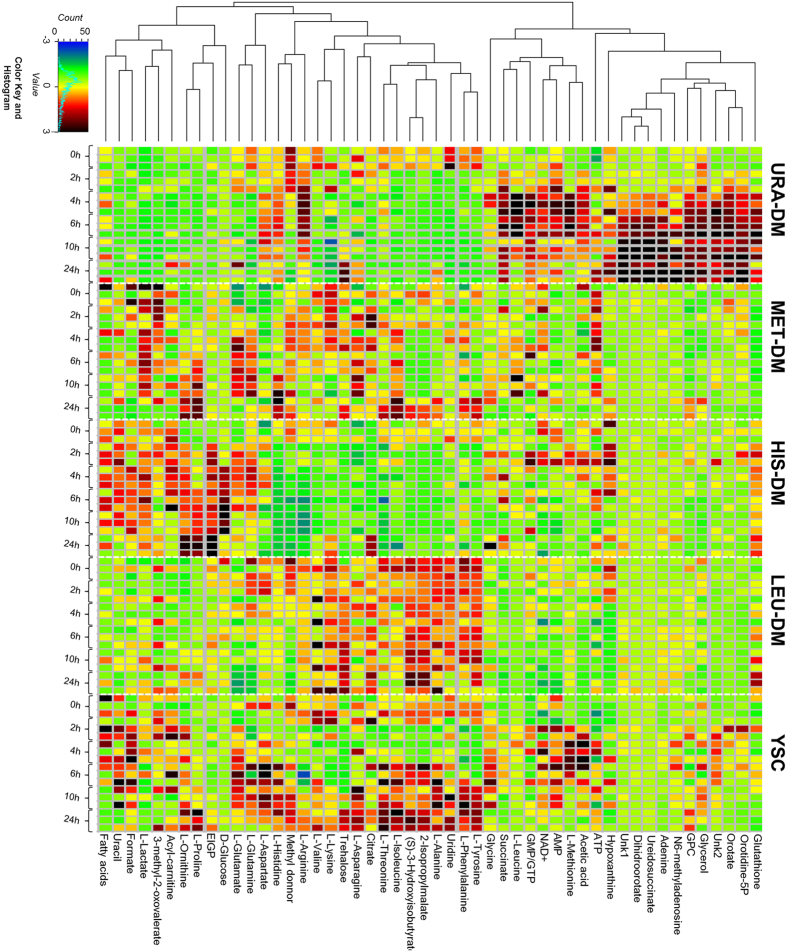
Heatmap of the auto-scaled concentration estimates for all assigned metabolites. Metabolites were clustered using the Pearson method. All individual samples (including replicates) were included.

Hierarchical clustering of the auto-scaled concentration estimates defined three clusters: one corresponding to metabolites accumulated in the lack of uracil (Ura-DM), a second, less defined one, including metabolites accumulated in the lack of L-histidine (His-DM), and the last one including the remaining metabolites (Fig. 4). Close inspection of the individual profiles shows the non-consumption of metabolites in Leu-DM medium and quasi-cyclic variations for some metabolites (see for example L-methionine, 2-isopropylmalate and L-Tyrosine) in YSC and also for some of the auxotrophic starvation conditions tested.

### Metabolome variations during growth

**Figure 5:**
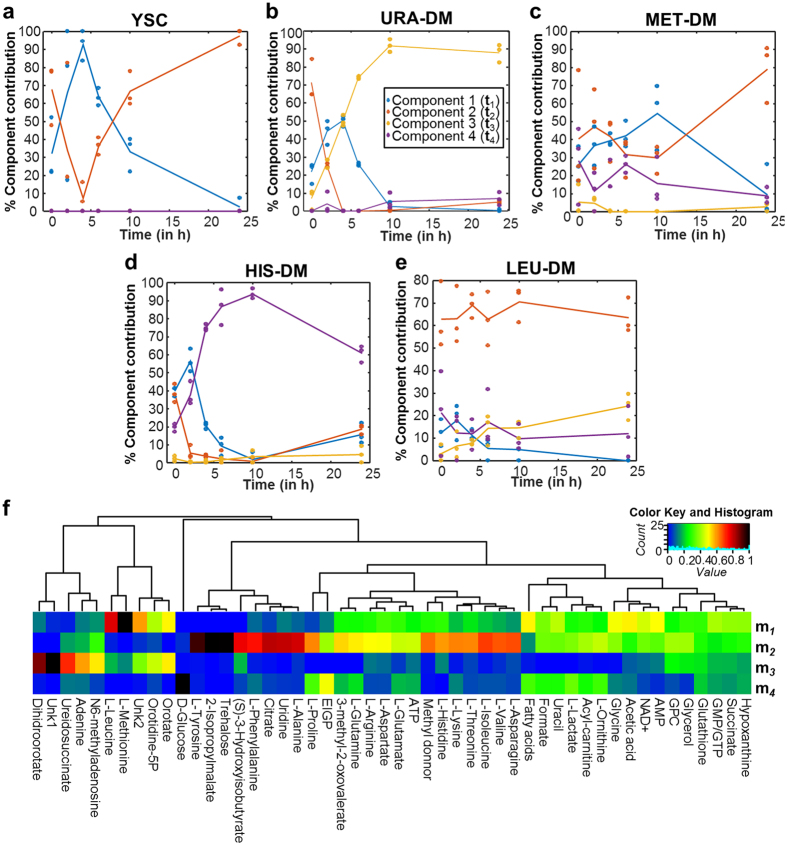
Growth pattern of yeast metabolism resolved by MCR-ALS using 4 components. (**a–e**) Temporal growth pattern (in %) of yeast cells cultured in YSC (**a**), URA-DM (**b**), Met-DM (**c**), His-DM (**d**) and Leu-DM (**e**) medium described by each MCR-ALS component. (**f**) Hierarchical clustering of the relative contribution of every metabolite in the 4 MCR-ALS resolved components given in **(a–e**) to every metabolite.

Estimated concentration changes from proton resonances were analyzed using MCR-ALS (see *Materials and Methods* and Supplementary Methods). Four temporal components, **t**_**1**_–**t**_**4**_, associated to four metabolic profiles, **m**_**1**_–**m**_**4**_, were obtained from this analysis, with an explained data variance of 85.7%. **t**_**1**_–**t**_**4**_ temporal components for each experimental condition are presented in Fig. 5a–e, whereas the **m**_**1**_–**m**_**4**_ metabolic profiles associated to each temporal profile are represented in the heatmap of Fig. 5f.

Most of the metabolic variability of the yeast metabolome during unrestricted growth (YSC, Fig. 5a) could be explained by only two MCR-ALS components (YSC, Fig. 5a). In addition, as observed in this figure, **t**_**1**_ and **t**_**2**_temporal components practically mirror one each other: Component **t**_**1**_ (blue dots and lines in Fig. 5a) peaked after 2–6 h of incubation, coinciding with the period of maximal growth, precisely the same time point at which component **t**_**2**_ (red dots and lines in Fig. 5a) showed a minimum. We thus assign the corresponding metabolic profiles (**m**_**1**_ and **m**_**2**_) to exponential and lag growth phases, respectively. Analysis of the metabolites associated to each of these two components revealed that **m**_**1**_ has strong contribution of L-methionine, L-leucine, fatty acids, uracil precursors (orotate and orotidine-5P), and AMP. On the other side, **m**_**2**_ shows strong contributions of amino acids and amino acid precursors (2-isopropylmalate and 3-hydroxyisobutyrate), citrate and trehalose, among others (Fig. 5f).

MCR-ALS results show completely different metabolic dynamic processes for each of the tested conditions, suggesting different patterns of arrest of cell growth depending on the missing auxotrophic marker. Samples grown in Leu-DM hardly showed any change in metabolic concentrations over the measured time, and their main contribution was for the temporal component **t**_**2**_, related to the stationary phase (Fig. 5e). In Ura-DM conditions (Fig. 5b), components **t**_**1**_ and **t**_**2**_ showed significant contributions only during the first two hours of incubation, whereas the specific temporal component **t**_**3**_ increased over time, peaking at 10 h and remaining as the major component for the rest of the analyzed period. A similar pattern was observed for His-DM samples (Fig. 5d), in which the specific component **t**_**4**_, peaked at 4–10 h, slightly decreasing afterwards. Analyses of the major metabolite contributors to the corresponding temporal profiles revealed a high contribution of precursors of uracil and also of other purine-related molecules to **m**_**3**_ and an equally strong contribution of D-glucose, erythro-imidazole-glycerol phosphate (EIGP), L-ornithine, L-proline and L-lactic acid to **m**_**4**_ (Fig. 5d). The contribution of the different components to the variability of Met-DM samples was more complex, with relevant contribution of **t**_**1**_, **t**_**2**_ and **t**_**4**_ during the first 10 h, followed by a strong increase of the stationary phase-related component **t**_**2**_, which became predominant at the end of the incubation (Fig. 5c).

**Figure 6:**
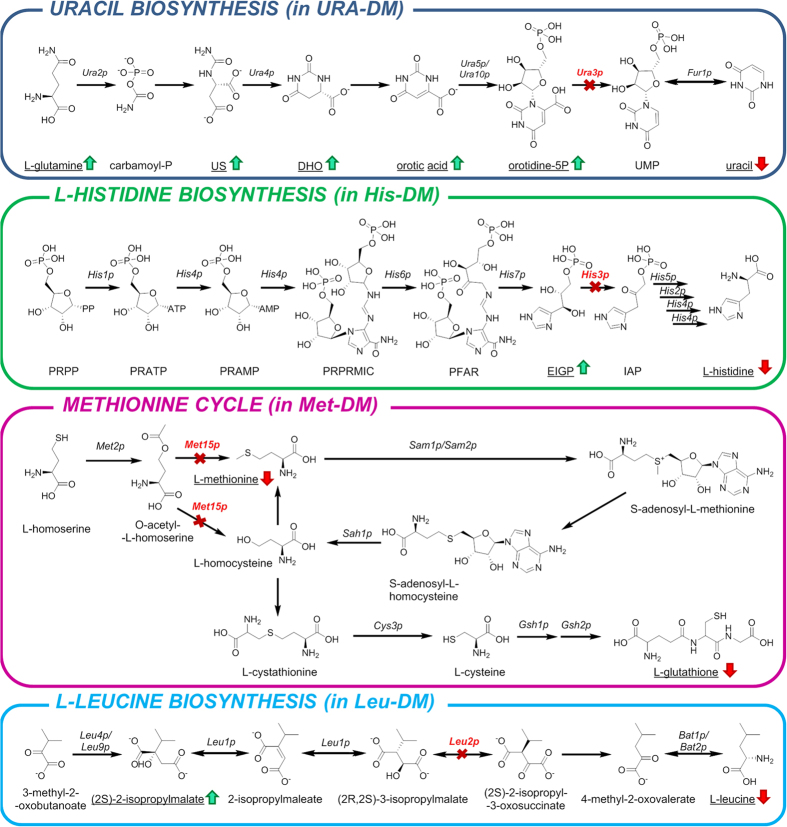
Disrupted metabolic pathways of the used auxotrophic yeast strain. Coloured arrows denote the accumulation (green) or consumption (red) of the detected metabolites in the associated DM medium. Red enzyme names and red crosses denote the depleted genes. Underlined metabolites were those detected and quantified by ^1^H NMR. US: ureidosuccinate; DHO: dihydroorotate; UMP: uridine monophosphate; PRPP: 5-phospho-α-D-ribose PP; PRATP: 5-phospho-α-D-ribose ATP; PRAMP: 5-phospho-α-D-ribose AMP; PRPRMIC: 1-(5-phospho-β-D-ribosyl)-5-[(5-phosphoribosylamino) methylideneamino]imidazole-4-carboxamide; PFAP: phosphoribulosylformimino-AICAR-P; EIGP: D-erythro-imidazole-glycerol-phosphate; IAP: imidazole-acetol-phosphate.

Starvation-induced metabolomic changes can be interpreted under the point of view of the biochemical pathways interrupted by the corresponding gene disruptions (Fig. 6). This analysis reveals a decrease in concentration of metabolites downstream the disrupted gene under all four starvation conditions and a parallel increase of upstream genes in at least three of them (Uracil, L-histidine and L-leucine). Note that these changes were condition-specific, that is, they only affected the metabolic pathway related to each particular starvation condition. Therefore, these data suggest a specific regulation for each metabolic pathway, at least at the metabolic level. Other condition-specific changes indicate far-ranging effects of the auxotrophic starvation. For example, different purine-related metabolites (i.e. AMP, hypoxanthine and N^6^-methyladenosine) were also accumulated in Ura-DM, suggesting that both purine and pyrimidine pools were affected due to the lack of uracil in the medium. N^6^-methyladenosine, which is the most common internal mRNA modification in eukaryotes^26^, was first detected in yeast under sporulation conditions^27^. The increasing presence of this compound in the Ura-DM extracts suggests that the lack of uracil induces mRNA degradation, likely to increase the diminishing uracil reservoirs to enhance cell survival under this limiting condition. Another example of the effects of auxotrophic starvation upon apparently unrelated metabolic pathways is the increase of intracellular D-glucose in histidine-starving cells, the meaning of which is unclear at the present.

## Discussion

There are two characteristics of ^1^H NMR analysis that limit its use for metabolomic studies. On one hand, it is assumed that in terms of sensitivity (limit of detection), NMR clearly lags behind chromatographic/MS-based methods. Second, the intrinsic complexity of the ^1^H NMR spectra due to overlapping makes very complicate to evaluate complex mixtures of a wide variety of molecules present at very different molar concentrations, such as in metabolite extracts. Although the sensitivity and the resolution aspect is an instrumental problem that can be tackled by many technical improvements (higher magnetic fields, low temperature probes, etc.), the dynamic range problem^28^ in NMR still prevents the detection of trace compounds. Despite these limitations, NMR is a convenient choice for performing screening metabolomics-based studies, since the compounds present in the studied samples can be unambiguously identified and robustly quantified due to the inherent particularities of this technique.

In this manuscript, we applied PCA and ASCA to first analyze the raw ^1^H NMR spectral dataset, and MCR-ALS on the metabolite concentrations. With these three chemometric methods, the metabolic responses of yeast at four starving conditions have been characterized (summarized in Table 1). Finally, we used all this information to interpret the observed variations in a biochemical context.

Chemometric analysis of the complete ^1^H NMR spectral dataset detected specific variations in yeast metabolomic profiles as a response to different auxotrophic starvation conditions, without requiring the identification of the metabolites implicated in these changes. The analysis allows drawing some a priori unexpected conclusions, like that single amino acid starvation (e.g., DM-Leu and DM-His samples) may trigger very different responses in the yeast metabolome, or that yeast growth efficiency is not correlated to the metabolic variance. These conclusions can be easily deduced from Figs 1 and 2, simply by comparing growth curves with PCA score plots for the different growing conditions. The same analysis reveals that metabolomic differences between dropout and control cultures tended to increase over time, and that this divergence is maximal in the case of Ura-DM samples. In contrast, Met-DM and Leu-DM samples showed relatively little changes during the 24-hour incubation period, compared to the other two drop-out media and even to the control samples (Fig. 3). On the other hand, the interaction between the two factors, *time* and *yeast medium*, confirmed by ASCA, indicates that the lack of auxotrophic markers alters the velocity and duration of the different yeast growth events.

Therefore, either by using PCA or ASCA, the analysis of ^1^H NMR data allowed the characterization and evaluation of the physiological conditions of a cultured organism. Qualitative metabolite concentration changes can be extracted from the loading plots associated to PCA analysis, as shown elsewhere^29^, although they can be also observed by a simpler heatmap representation, as in Fig. 1.

We interpret the metabolic changes observed in yeast under unrestricted conditions (components **m**_**1**_ and **m**_**2**_) as reflecting the alternation between the stationary and the exponential growth phases, induced by the presence of high concentrations of glucose in the medium and regulated by the PP2A/TOR signalling pathway^30^. These results are also consistent with the known relative decrease of oxidative metabolism (including respiration) in yeast at high glucose conditions^31^. The enhanced growth observed in the exponential phase is characterized by a decrease in the amino acid pools (**m**_**2**_ in Fig. 5f) and with an increase of the transcription machinery (**m**_**1**_ in Fig. 5f). On the other side, the metabolic transition at the beginning of the stationary phase (10 h and 24 h) is marked by the increase of the levels of trehalose, a resistance metabolite, and of some amino acids (**m**_**2**_ in Fig. 5f). This is probably caused by a decrease in the translation activity and linked to the restoration of the internal amino acid pools^32^,^33^.

Similarly, the metabolic variations associated to the corresponding auxotrophic starvation (components **m**_**3**_ and **m**_**4**_) can be easily interpreted as the primary metabolic response from depleting the missing metabolites (i.e. uracil in Ura-DM), consisting on the accumulation of the precursors generated at the metabolic steps immediately upstream them (i.e. EIGP to His3p and Orotidine-5P to Ura3p), (Fig. 6). It is important to note that this analysis allowed as well the detection of a priori unexpected metabolome variations, like the purines accumulation after inducing pyrimidine (uracil) starvation, or the accumulation of D-glucose in histidine-starving conditions.

It is important to note that the use of MCR-ALS for studying -omic data should be applicable to essentially any other biological sample aside of the model organism *Saccharomyces cerevisiae*. This includes population mixtures or non-model biological organisms in which conventional pathway analyses approaches (i.e. ‘fluxomics’) might be difficult to implement.

Perhaps the most streaking observation of the metabolome analysis was the radical differences between the effects of L-histidine starvation on one side and of L-leucine and L-methionine starvation on the other side. Irrespectively of the effects for the global growth, Leu-DM and Met-DM cultures showed minimal metabolome variations during the 24 h-incubation period (actually, even less variations than cells cultured under unrestricted conditions), whereas His-DM cells’ metabolome steadily diverged from its initial composition upon time. This effect, observed both for the complete ^1^H NMR spectral profiles and when identified metabolites were individually analyzed, suggests different regulatory pathways activated as a response to the different amino acids. Both L-leucine and L-methionine starvation have been linked to G0/G1 arrest through the PP2A/TOR signaling pathway, a mechanism fully compatible with our observations at the metabolome levels^34^,^35^,^36^,^37^,^38^.

In this work, we used a genetically well-known model system to test the power of ^1^H NMR to analyze metabolome effects of enzymatic pathway disruptions without any previous hypothesis nor anticipation of the nature of the possible metabolic pathway blockage. Analysis of the complete ^1^H NMR spectral profiles allowed us to distinguish between those conditions blocking the entry into the cell cycle (Leu-DM and Met-DM) from those that allow the progression of at least some metabolic pathways (e.g., Ura-DM and His-DM), and this without the need of identifying the specific metabolic concentration changes. Identification of the altered metabolites under each starvation condition could in principle allow the identification of the affected metabolic pathway in all four cases, and pinpoint the disrupted enzymatic steps in at least two of them. We conclude that the proposed NMR metabolomics strategy can be useful for studying models of metabolomic disruption either with genetic defects or with enzymatic inhibitors in many biological systems, including the study of the molecular target of biocides or the metabolic response of malignant cells to antitumorals.

Therefore, we consider that the synergy between NMR metabolomics and molecular biology can be very useful in genetic studies, such as transcriptomics, but may be also applicable in genetic diagnostics and drug development.

## Methods

### Yeast Growth

*S. cerevisiae BY4741* (MATa; his3Δ1; leu2Δ0; met15Δ0; ura3Δ0) cells were pre-cultured in YPD (1% yeast extract, 1% peptone, 2% glucose) medium on an orbital shaker (150 rpm) at 30 °C overnight. All following cultures were cultured with these shaking and temperature conditions. 2 L of YNB Synthetic Complete medium (YSC, 1.7 g/L Yeast Nitrogen Base without amino acids and sulphate (Difco), 5 g/L (NH_4_)_2_SO_4_) supplemented with the appropriated auxotrophic markers (4 mg/L uracil, 1 mg/L leucine, 1 mg/L histidine, and 1 mg/L methionine, as requested) were inoculated with 200 μl of the pre-culture sample and left at the same temperature and shaking conditions until the culture reached an optical density at 600 nm (OD_600_) of approximately 0.8–1. Pellets from these resulting cultures were collected by centrifuging the cultures, but not washed, at 2000 rpm for 3 min and 4 °C. Pellets were used right after for inoculating erlenmeyers containing either YSC medium (control) or drop-out media missing either L-leucine, L-histidine, uracil or L-methionine, up to a final OD_600_ of 0.4–0.5. Resulting cultures were grown at 30 °C and 150 rpm.

### Sample collection

100 ml aliquots of every culture were collected six times during one day (0 h, 2 h, 4 h, 6 h, 10 h and 24 h). Samples were arrested with a cold shock in ice and cell were harvested by centrifugation at 4000 g for 3 min, discarding the supernatant. Cells were washed twice in Na_2_HPO_4_ 100 mM pH 7.0 followed by a centrifugation at 4700 g for 3 min. Resulting pellets were stored at −80 °C and lyophilized. Cell density was calculated as OD_600_, and viable cell counted by plating culture dilutions in YPD agar plates (1% yeast extract, 1% peptone, 2% agar, 2% glucose) for each sample.

### Metabolite extraction

Metabolites were extracted by following the protocol published in a previous work^20^. 1800 μl of a solution of methanol-chloroform 1:2 (4 °C) were added to the pellet, followed by a vigorous vortexing. A cold shock is then applied to the pellets for 5 times using the following procedure: the pellets are submerged in liquid nitrogen for 1 minute and consequently thawing in ice for 2 minutes. 400 μl of water are added to create the biphasic system. After homogenization by vortexing, a 3 min centrifugation at 16500 rpm and 4 °C is carried out. The aqueous phase (upper part) is collected. This process is repeated and water and methanol are removed from the aqueous phase in a speedvac.

### NMR sample preparation

Aqueous samples were dissolved in 700 μl of deuterated phosphate buffer (Na_2_DPO_4_ 100 mM, pH 7.0) in D_2_O with DSS 0.2 mM as internal standard.

### ^1^H NMR experiments

Spectra were recorded in a 400 MHz Varian spectrometer, using a spectrometer frequency of 400.14 MHz with a OneNMR Probe and a ProTune System (Agilent). Spectral size range covered from −2 to 10 ppm. Receiver gain was fixed to 34. Also, 512 scans were used with a relaxation delay of 5 seconds. Spectral size contained 65 k data points, and the acquired size was made of 32 k complex data points.

### NMR spectra preprocessing

Spectra were preprocessed with MestreNova v.10.0 (Mestrelab Research, Spain). Spectra preprocessing consisted in an exponential apodization of 0.5 Hz, a manual phasing and a baseline correction with Bernstein polynomial of 3rd order. After adjusting the reference to DSS, water (4.41–5.16 ppm), methanol (3.30–3.37 ppm), chloroform (7.64−7.69 ppm) and DSS (<0.8 ppm) regions were removed. Data points which chemical shifts were higher than 10.3 ppm were also removed. The final NMR dataset consisted on a data matrix of 90 spectra (rows) having 35,342 ppm values (columns) each one. This data matrix was stored in ASCII file format.

### Metabolite identification

Metabolite assignment was performed by a detailed targeted metabolite profiling analysis of the ^1^H NMR signals using a home-made ^1^H NMR spectra library^20^ and also the Yeast Metabolome Data Base library^39^ (YMDB). Proton correlations were checked on gCOSY spectra. Additional metabolite confirmations were performed using complementary NMR pulse sequences (see Supplementary Methods). Pathway diagram data was obtained from *Biocyc* database^40^.

### Metabolite quantification

Relative metabolite quantifications of the ^1^H NMR spectral matrix were performed using BATMAN R^41^ package. Further information about how Batman works and the exact protocol can be found elsewhere^20^,^42^.

### Chemometric data analysis of the NMR dataset

^1^H NMR spectra from the previous preprocessing steps were imported to Matlab R2014a (The Mathworks Inc. Natick, MA, USA) and analysed with the PLS toolbox 7.8.0 (Eigenvector Research Inc., Wenatchee, WA, USA). In order to eliminate sample size effects, ^1^H NMR spectra were normalized using the Probabilistic Quotient Normalization (PQN)^43^ method, taking only into account the region of 0.8–3.8 ppm. A reference spectrum was used for every time and tested condition, consisting on the average of all spectra measured at the same conditions in the previous sample collection time, except for time 0 h spectra, where the reference spectrum used was obtained using all samples measured at 0 h, regardless of the tested condition studied.

Chemical shift corrections were performed using the icoshift^44^ algorithm in the 7.02–7.14, 7.72–7.77, 8.51–8.55, 8.56–8.63 and 9.09–9.17 ppm regions. For Principal Component Analysis (PCA) and Analysis of variance of Simultaneous Component Analysis (ASCA), the 7.78–8.00 region was ignored.

PCA was applied to the mean-centered PQN-normalized NMR spectral data matrix (dimensions of 90 × 34443 data points), using Cross-Validation with Venetian Blinds to find the most reliable number of components to be included in the models.

ASCA^45^,^46^ was applied to the NMR spectral dataset to evaluate the effects of each experimental factor (*time* and *medium* in this study). In order to check the statistical significance of the effects of the investigated factors and of their possible interactions, a permutation test was performed^47^,^48^. In this study, the number of permutations was set at 10000. Before performing ASCA, matrix dimensions were reduced by taking every 1 out of 10 values, thus obtaining a reduced size data matrix with 90 × 3445 values. In contrast to PCA, data used were not mean-centered.

### Chemometric data analysis of the concentration profiles

In order to resolve the metabolic patterns that evolve over time, the Multivariate Curve Resolution by Alternating Least Squares^49^ (MCR-ALS) method has been used. Data analysis was performed using the MCR-ALS GUI 2.0^23^ under Matlab 2014b (The Mathworks Inc. Natick, MA, USA) environment.

MCR-ALS is a chemometric method which decomposes a data matrix using the following bilinear model:


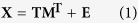


In this particular case, the data matrix **X** (size I × J) has the concentrations of the J metabolites obtained by integrating their corresponding proton resonances, in the I yeast samples, cultured in a particular medium during a particular time period. This matrix bilinear decomposition gives two factor matrices, **M**^**T**^ and **T**, the matrix of metabolic profiles, **M**^**T**^ (N × J), and the matrix of temporal profiles, **T** (I × N). Each metabolic profile in **M** shows its metabolite composition, whereas **T** shows the contribution of each individual metabolic profile in every sample at different collection times. N represents the number of components used in the decomposition generated in the MCR-ALS analysis. N can be initially estimated by using a singular value decomposition (SVD)^50^. **E** matrix (size I × J) contains the residual information not explained by the model using the N considered components. A more detailed description of this method can be found in Supplementary Methods.

The quality of the MCR-ALS model was measured evaluating the lack-of-fit parameter, which is expressed by the percent of explained variance (R^2^)^23^.

On the other hand, hierarchical cluster analysis of concentration estimates and of the **M** matrices was performed. Clustering was performed using the heatmap.2 function from the gplots R package^51^ using the complete agglomeration method for clustering. Metabolite concentration estimates were auto-scaled before analysis, whereas heatmap representations of **M** matrices had the MCR-ALS results without any data pretreatment.

## Additional Information

**How to cite this article**: Puig-Castellví, F. *et al*.^1^H NMR metabolomic study of auxotrophic starvation in yeast using Multivariate Curve Resolution-Alternating Least Squares for Pathway Analysis. *Sci. Rep*. **6**, 30982; doi: 10.1038/srep30982 (2016).

## Supplementary Material

Supplementary Information

Supplementary Table S1

## Figures and Tables

**Table 1:** Summary of the responses observed in starved yeast cultures.

Starving condition	Related gene	Cell growth (OD600)	Metabolic variation over time (PCA)	Effect in cell growth timing **(ASCA)**	Metabolic response (MCR-ALS)
Uracil	URA3	153%	↑↑	YES (p ≤ 0.0001)	Enhanced biosynthesis of uracil precursors and purine-related compounds (m_3_ metabolic profile)
L-methionine	MET15	197%	↑	YES (p = 0.0180)	Prolongation of log phase
L-histidine	HIS3	184%	↑↑	YES (p ≤ 0.0001)	Enhanced biosynthesis of L-histidine precursors (m_4_ metabolic profile)
L-leucine	LEU3	140%	—	YES (p ≤ 0.0001)	Cell cycle arrest
