# Supplementary material for: 1H NMR metabolomic study of auxotrophic starvation in yeast using Multivariate Curve Resolution-Alternating Least Squares for Pathway Analysis
Source: Sci Rep. 2016 Aug 3;6:30982. doi: 10.1038/srep30982 (PMC4971537; doi:10.1038/srep30982)
Supplement: Supplementary Information [file srep30982-s1.pdf]

^{1}H NMR metabolomic study of auxotrophic starvation in yeast using Multivariate Curve Resolution-Alternating Least Squares for Pathway Analysis

Francesc Puig-Castellví[a], Ignacio Alfonso[b], Benjamín Piña[a] & Romà Tauler[a]*

[a] *Department of Environmental Chemistry, Institute of Environmental Assessment and Water Research (IDAEA-CSIC), Jordi Girona 18-26, 08034 Barcelona, Catalonia, Spain;* [b] *Department of Biological Chemistry and Molecular Modelling, Institute of Advanced Chemistry of Catalonia (IQAC-CSIC), Jordi Girona 18-26, 08034 Barcelona, Catalonia, Spain*

*Correspondence: roma.tauler@idaea.csic.es

TABLE OF CONTENTS

Supplementary Methods 2

Supplementary Figures and Tables with captions 5

Supplementary Methods

Yeast Growth. For assignment purposes, *S. cerevisiae BY4741* (MATa; his3Δ1; leu2Δ0; met15Δ0; ura3Δ0) cells were cultured in 1-L of His-DM medium on an orbital shaker (150 rpm) at 30 $^{\circ}$C for 24 h.

Metabolite extraction on the extract from 1 L culture of His-DM. The same method as in the 100-ml samples case was applied, but the solvent volumes were up-scaled accordingly.

NMR experiments for assignment confirmation. In order to check proton correlations and to provide a more robust assignment, additional homonuclear (gCOSY, zTOCSY and zTOCSY1D) and heteronuclear experiments (^{1}H/^{13}C gHMBCAD and ^{1}H/^{13}C gHSQCAD) were performed. Unless stated, receiver gain was fixed to 34. In all bidimensional experiments, 512 t1 increments were used. Both zTOCSY and gCOSY experiments were acquired with 8 scans. zTOCSY1D experiments were acquired with 256 scans. In zTOCSY and zTOCSY1D, spinlock time used was 80 ms. Heteronuclear experiments were recorded using a relaxation delay of 1 second and an automatic detection of the optimum value for the receiver gain. ^{1}H/^{13}C gHMBCAD and ^{1}H/^{13}C gHSQCAD were recorded using 16 scans. The carbon spectral size covered from -10 to 190 ppm.

Metabolite identification. Proton correlations were checked on gCOSY spectra. 2-isopropylmalate assignment was confirmed using zTOCSY1D, irradiating individually the two resonances from each methyl, with a window width of 18 Hz for each doublet. Results from these experiments are in agreement with spectroscopic values of the isopropyl branch of the 2-isopropylmalate, as seen in YMDB. On the other hand, D-eritro-imidazole-glycerol-phosphate was confirmed by contrasting spectroscopic data from ^{1}H/^{13}C gHMBCAD and ^{1}H/^{13}C gHSQCAD of a metabolic yeast extract from a 1L culture for 24 h in His-DM.

Chemometric data analysis of the concentration profiles. MCR-ALS was applied to the whole dataset (**X**, size dimension of 90 x 50), and to the subset containing only samples cultured in YSC medium ($\mathbf{X}_{\mathbf{YSC}}$, size dimension of 18 x 50). An initial estimation of either **T** or **M** factor matrices is also needed to start the MCR-ALS analysis. For the MCR-ALS decomposition of

$\mathbf{X}_{\mathbf{YSC}}$, these initial estimations were selected from the purest samples (rows)[1,2]. MCR-ALS results of this subset, using two components, are given in the **Supplementary Fig. S3**. In this analysis, R^2 value was 84.6.

In the MCR-ALS analysis of the whole dataset, **X**, metabolic profiles obtained in previous MCR-ALS analysis ($\mathbf{M}_{\mathbf{YSC}}$) were used as the initial estimates of the growth components. Initial estimations of component profiles related to starving conditions were normalized concentration estimates of original samples containing an important contribution due to stress. In this case, these two samples corresponded to samples cultured in Ura-DM and His-DM, and collected after 10 h. Only additional components related to Uracil- and Histidine-starving conditions were added in the MCR-ALS analysis of the whole dataset since it had been observed in the PCA analysis that the major variability derived from these two cultures, and due to the fact that only these two starving conditions triggered the synthesis of the specific metabolite precursors, as showed in the heatmap of **Figure 4**.

MCR-ALS resolution using five components (three components related to growth at normal conditions and two related to growth at starving conditions) was also evaluated. However, when five components were used, the exponential phase ($\mathbf{t}_1$) was divided in two peaks and we considered that explaining the exponential phase with two components was less meaningful. In addition, when five components were used instead of four, lack-of fit and R^2 do not considerably improve. These values were 34.6 % and 88.0%, respectively, for five components used. For four components, lack-of-fit and explained variance corresponded to 37.5 % and 85.7 %, respectively.

Then, the estimation of **T** and **M** factor matrices was performed by means of the alternating least squares optimization under constraints. In this work, we applied non-negativity constraints on both factor matrices and an equal height constraint on **M** matrix. Metabolic profiles related to starvation metabolism were constrained to not be included in the resolution of control samples[3].

Since **T** and $\mathbf{M}^T$ matrices are obtained using an iterative least squares process with the goal of explaining the maximum possible variance, metabolites at higher concentration would have a higher weight in the model. In order to avoid that, **X** raw data matrix was scaled by the total sum of every column (metabolite concentrations) before MCR-ALS analysis. With this approach, low concentrated metabolites (for all the samples) become equally represented than the high concentrated ones.

References:

1. Windig W, Stephenson DA (1992) Self-modeling mixture analysis of second-derivative near-infrared spectral data using the SIMPLISMA approach. Analytical Chemistry 64: 2735-2742.
2. Windig W, Guilment J (1991) Interactive self-modeling mixture analysis. Analytical Chemistry 63: 1425-1432.
3. Tauler R, Smilde A, Kowalski B (1995) Selectivity, local rank, three-way data analysis and ambiguity in multivariate curve resolution. Journal of Chemometrics 9: 31-58.

Supplementary Figures and Tables with captions

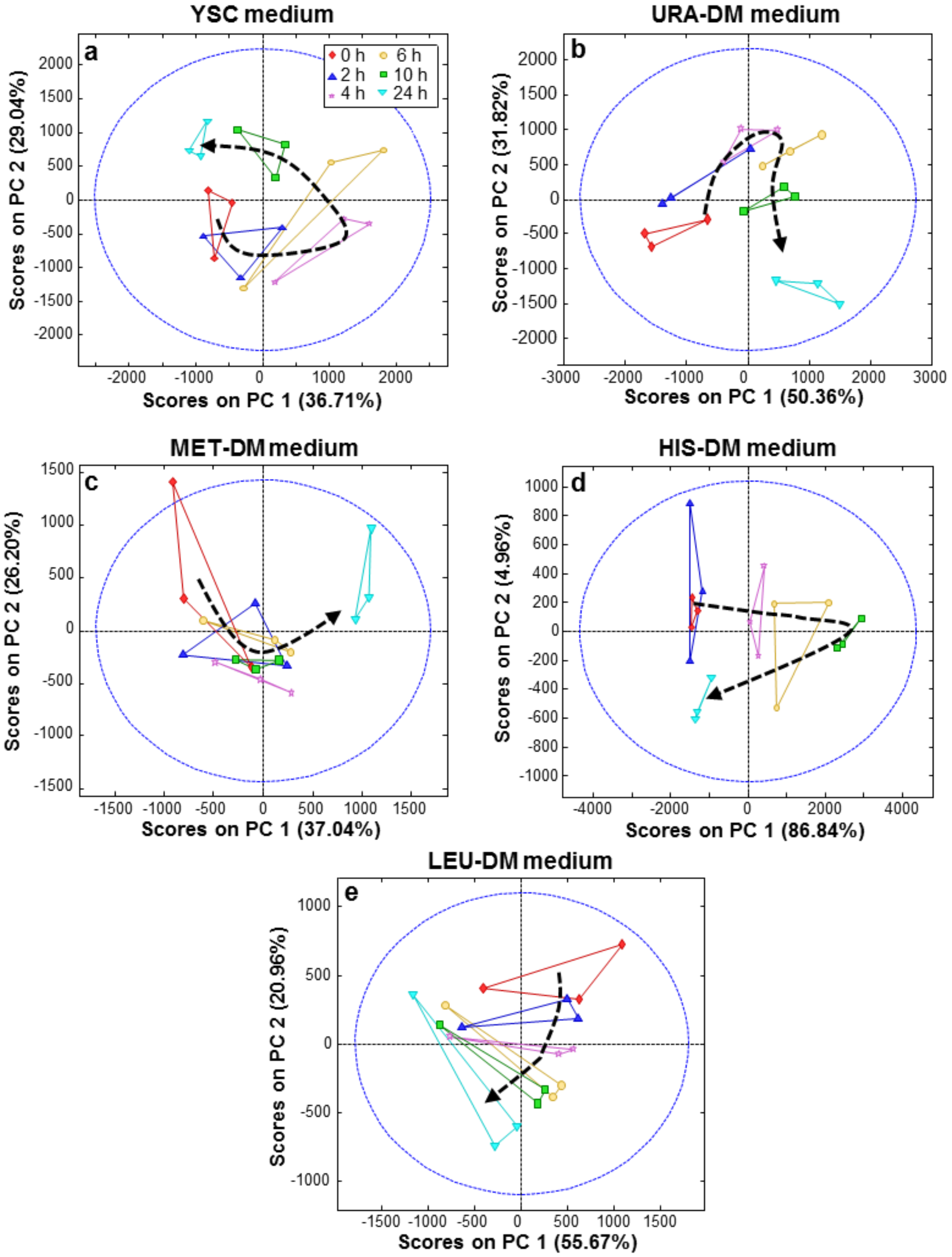

Figure S1. PCA analysis of the internal metabolic variance of starved cultures over time. a-e) PCA scores projection on PC1-PC2 subspace of samples cultured in YSC **(a)**, Ura-DM (**b**), Met-DM (**c**), His-DM (**d**) and and Leu-DM (**e**).

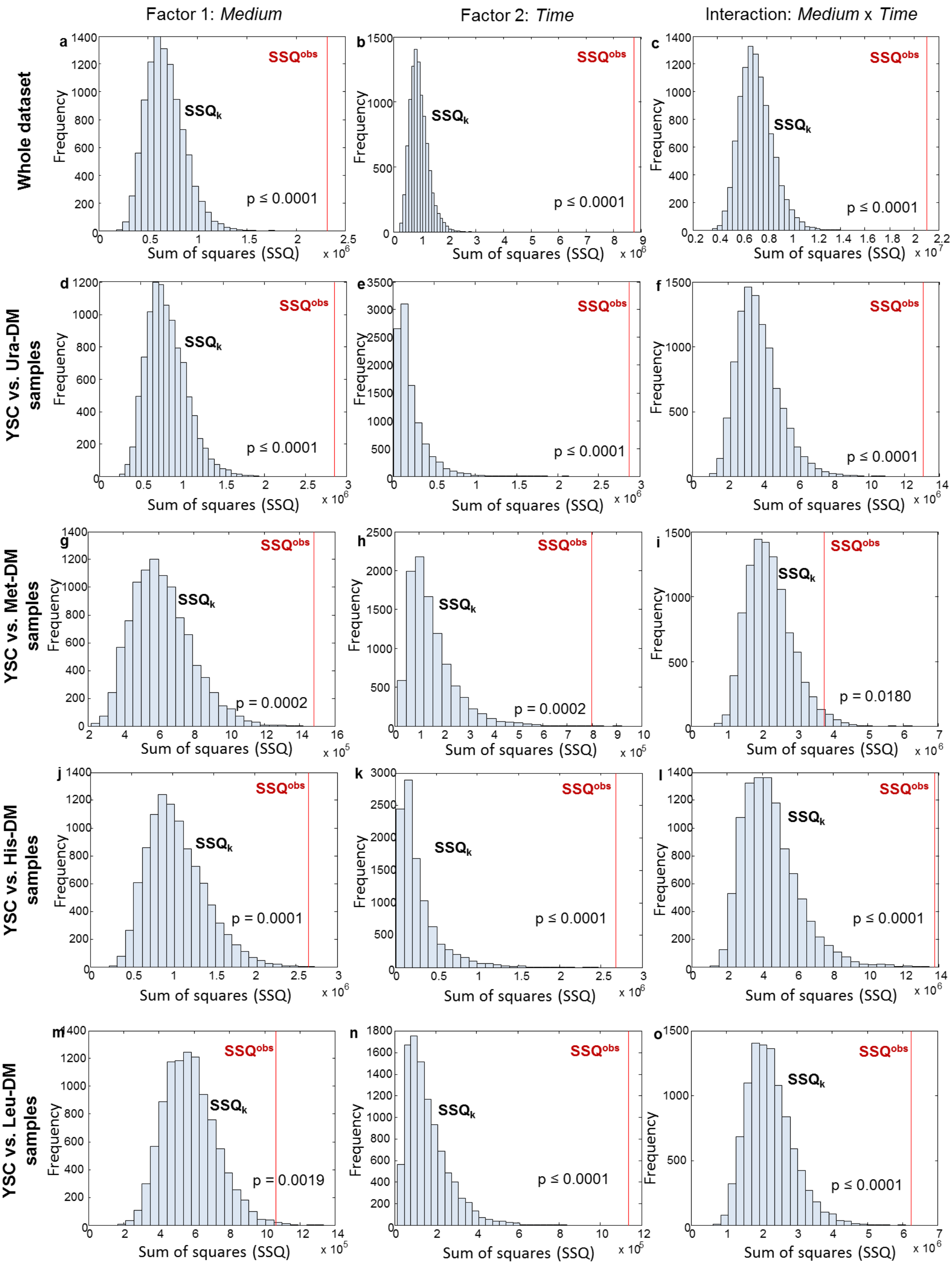

Figure S2. Histogram of the sum of squares (SSQ) obtained during permutation test in ASCA analyses. **a-c**) All samples. **d-f**) YSC vs. Ura-DM samples. **g-i**) YSC vs. Met-DM samples. **j-l**) YSC vs. His-DM samples. **m-o**) YSC vs. Leu-DM samples. **a**), **d**), **g**), **j**) and **m**) histograms show the significance of the *Medium* factor; **b**), **e**), **h**), **k**) and **n**) histograms show the significance for the *Time* factor; and **c**), **f**), **i**), **l**) and **o**) histograms show the significance for the *Medium* x *Time* interaction. The number of

permutations, k, used was 10000. Factors (**a** and **b**) or their interaction (**c**) are considered significant, as the sum of squares for each observed value (SSQ^{obs}) are larger than 95 % of the SSQ values obtained when the corresponding levels are randomized. For more information about the permutation test, see Zwanenburg et al. (2011).

Table S1. Metabolite assignment. ^{1}H NMR spectroscopic data (chemical shift, multiplicity, proton integral, and coupling constant) of the assigned metabolites. ^{13}C spectroscopic data is also provided for the erythro-imidazole-glycerol phosphate compound. CAS, HMDB, YMDB and KEGG codes are provided when existing.

#	Metabolite name	^{1}H and ^{13}C NMR signals assigned	CODE			
			CAS	YMDB	HMDB	KEGG
1	Acetic acid	1.90 ppm (s, 3 H)	64-19-7	YMDB00056	HMDB00042	C00033
2	Acyl-carnitine	3.19 ppm (s, 9 H)	25518-54-1	YMDB01529*	HMDB02250*	C02301
3	Adenine	8.18 ppm (s, 1 H); 8.23 ppm (s, 1 H)	73-24-5	YMDB00887	HMDB00034	C00147
4	AMP	6.13 ppm (d, J=6.0 Hz, 1 H); 8.26 ppm (s, 1 H); 8.59 ppm (s, 1 H)	61-19-8	YMDB00097	HMDB00045	C00020
5	ATP	6.14 ppm (d, J=5.1 Hz, 1 H); 8.53 ppm (s, 1 H); 8.26 ppm (s, 1 H)	56-65-5	YMDB00109	HMDB00538	C00002
6	Citric acid	2.50 ppm (s, 0.66 H); 2.54 ppm (s, 1.33 H); 2.63 ppm (s, 1.22 H); 2.67 ppm (s, 0.70 H)	77-92-9	YMDB00086	HMDB00094	C00158
7	EIGP	δ_H: 7.17-7.25 ppm (s, 1 H); 7.83-8.06 ppm (s, 1 H); 4.78 ppm (nd, 1 H); 3.99 ppm (nd, 1 H); 3.77 ppm (nd, 1 H); 3.62 ppm (nd, 1 H)<br>δ_C: 65.1 ppm (CH_2), 69.5 ppm (CH), 76.3 ppm (CH), 119.8 ppm (CH), 138.6 ppm (CH)	36244-87-8	YMDB00089	HMDB12208	C04666
8	D-Glucose	3.24 ppm (dd, J=(7.8 Hz, 9.2 Hz), 0.71 H); 3.35-3.56 ppm (mm, 2.63 H); 5.22 ppm (d, J=3.8 Hz, 0.36 H)	50-99-7	YMDB00286	HMDB00122	C00031
9	L-dihydroorotic acid	2.76 ppm (d, J=6.4 Hz, 0.43 H); 2.81 ppm (d, J=6.4 Hz, 0.57 H)	5988-19-2	YMDB00396	HMDB03349	C00337
10	Methyl donnor (R-S-CH_3)	2.10 ppm (s, 3 H)	75-18-3	-	HMDB02303	-
11	Fatty acid singlet	1.24 ppm (s, 4 H)	143-07-7	YMDB00678**	HMDB00638**	C00162
12	Formic acid	8.44 ppm (s, 1 H)	64-18-6	YMDB00385	HMDB00142	C00058
13	Glutathione	2.15 ppm (q, J=7.6 Hz, 2 H); 2.87-3.00 ppm (mm, 2 H); 4.56 ppm (dd, J=(7.0 Hz, 5.2 Hz), 1 H)	70-18-8	YMDB00160	HMDB00125	C00051
14	Glycerol	3.51-3.58 ppm (mm, 2.07 H); 3.61-3.67 ppm (mm, 2.12 H); 3.77 ppm (tt, J=(6.5 Hz, 4.4 Hz), 0.79 H)	56-81-5	YMDB00283	HMDB00131	C00116
15	Glycerophospho-choline	3.22 ppm (s, 9 H); 3.56-3.71 ppm (mm, 4 H); 4.27-4.36 ppm (mm, 2 H)	28319-77-9	YMDB00309	HMDB00086	C00670
16	Glycine	3.55 ppm (s, 2 H)	56-40-6	YMDB00016	HMDB00123	C00037
17	GMP/GTP	5.93 ppm (d, J=5.3 Hz)	85-32-5/ 86-01-1	YMDB00261/ YMDB00558	HMDB01397/ HMDB01273	C00144/ C00044
18	S-3-Hydroxyisobutyric acid	1.06 ppm (d, J=6.9 Hz)	2068-83-9	YMDB00337	HMDB00442	C06001
19	Hypoxanthine	8.18 ppm (s, 1 H); 8.20 ppm (s, 1 H)	68-94-0	YMDB00555	HMDB00157	C00262
20	2-Isopropylmalic acid	0.84 ppm (d, J=6.9 Hz, 3 H); 0.89 ppm (d, J=6.9 Hz, 3 H)	49601-06-1	YMDB00106	HMDB00402	C02504
21	L-alanine	1.47 ppm (d, J=7.1 Hz, 3 H); 3.76 ppm (q, J=7.2 Hz, 1 H)	56-41-7	YMDB00154	HMDB00161	C00041
22	L-arginine	1.58 - 1.79 ppm (ms, 2 H); 1.80-2.00 ppm (mm, 2 H); 3.23 ppm (t, J=6.9 Hz, 2 H); 3.76 ppm (t, J=6.1 Hz, 1 H)	74-79-3	YMDB00592	HMDB00517	C00062
23	L-asparagine	2.82 ppm (d, J=7.6 Hz, 0.286 H); 2.86 ppm (d, J=7.6 Hz, 0.714 H)	70-47-3	YMDB00226	HMDB00168	C00152
24	L-aspartic acid	2.64 ppm (d, J=8.9 Hz, 0.33 H); 2.69 ppm (d, J=8.9 Hz, 0.67 H); 2.78 ppm (d, 3.7 Hz, 0.67 H); 2.83 ppm (d, J=3.7 Hz, 0.33 H); 3.89 ppm (dd, J=(8.8 Hz, 3.8 Hz), 1 H)	56-84-8	YMDB00896	HMDB00191	C00049
25	L-glutamic acid	1.99 - 2.17 ppm (ms, 2 H); 2.26-2.42 ppm (mm, 2 H); 3.75 ppm (dd, J=(7.2 Hz, 4.7 Hz), 1 H)	56-86-0	YMDB00271	HMDB00148	C00025
26	L-glutamine	2.44 (td, J = 7.5, 3.7 Hz, 1H)	56-85-9	YMDB00002	HMDB00641	C00064
27	L-histidine	3.11 ppm (d, J=7.4 Hz, 0.35 H); 3.15 ppm (d, J=7.7 Hz, 0.65 H); 7.06 ppm (s, 1 H); 7.81-7.92 ppm (s, 1 H);	71-00-1	YMDB00369	HMDB00177	C00135
28	L-isoleucine	0.93 ppm (t, J=7.4 Hz, 3 H); 1.000 ppm (d, J=7.1 Hz, 3 H)	73-32-5	YMDB00038	HMDB00172	C00407
29	L-lactic acid	1.31 ppm (d, J=7.0 Hz, 3 H); 4.10 ppm (q, J=6.9 Hz, 1 H)	79-33-4	YMDB00247	HMDB00190	C00186
30	L-leucine	0.95 ppm (d, J=6.1 Hz, 3 H); 0.95 ppm (d, J=6.1 Hz, 3 H)	61-90-5	YMDB00387	HMDB00687	C00123
31	L-lysine	1.35-1.60 ppm (mm, 2 H); 1.65-1.80 ppm (mm, 2 H); 1.81-1.94 ppm (ms, 2 H); 2.95-3.1 ppm (mm, 2 H); 3.75 ppm (t, J=6.1 Hz, 1 H)	56-87-1	YMDB00330	HMDB00182	C00047
32	L-methionine	2.13 ppm (s, 3 H); 2.63 ppm (t, J=7.5 Hz, 3 H)	63-68-3	YMDB00318	HMDB00696	C00073
33	L-ornithine	1.65-2.00 ppm (mm, 4 H); 3.04 ppm (t, J=7.6 Hz, 2 H)	70-26-8	YMDB00353	HMDB00214	C00077
34	L-phenylalanine	7.37 ppm (mm, 5 H)	63-91-2	YMDB00304	HMDB00159	C00079
35	L-proline	1.90-2.12 ppm (mm, 3 H); 2.27-2.40 ppm (mm, 1 H); 4.12 ppm (dd, J=(8.6 Hz, 6.4 Hz), 1 H)	344-25-2	YMDB00378	HMDB00162	C00148
36	L-threonine	1.32 ppm (d, J=6.6 Hz, 3 H); 3.57 ppm (d, J=4.9 Hz, 1 H); 4.19-4.28 ppm (mm, 1 H)	72-19-5	YMDB00214	HMDB00167	C00188
37	L-tyrosine	6.89 ppm (d, J=8.4 Hz, 2 H); 7.18 ppm (d, J=8.4 Hz, 2 H)	60-18-4	YMDB00364	HMDB00158	C00082
38	L-valine	0.98 ppm (d, J=7.1 Hz, 3 H); 1.03 ppm (d, J=7.1 Hz, 3 H); 2.26 ppm (mm, 1 H)	72-18-4	YMDB00152	HMDB00883	C00183
39	3-Methyl-2-oxovaleric acid	1.09 ppm (d, J=6.6 Hz, 3 H)	816-66-0	YMDB00168	HMDB00491	C00671
40	N^6-methyl-adenosine	2.96 ppm (s, 3 H); 6.11 ppm (d, J=4.4 Hz, 1H); 8.28 ppm (s, 1H); 8.29 ppm (s, 1H)	1867-73-8	-	HMDB04044	-
41	NAD^+	6.03 ppm (d, J=5.9 Hz, 1 H); 6.08 ppm (d, J= 5.3 Hz, 1 H); 8.16 ppm (s, 1 H); 8.17 ppm (d, J=6.1 Hz); 8.15-8.21 ppm (mm, 2 Hz); 8.42 ppm (s, 1H); 8.82 ppm (d, J=7.6 Hz, 1 H); 9.13 ppm (d, J=7.6 Hz, 1 H); 9.33 ppm (s, 1 H)	53-84-9	YMDB00110	HMDB00902	C00003
42	Orotic acid	6.18 ppm (s, 1 H)	65-86-1	YMDB00405	HMDB00226	C00295
43	Orotidine-5P	5.54 ppm (d, J=3.3 Hz, 1 H); 5.76 ppm (s, 1 H);	2149-82-8	YMDB00025	HMDB00218	C01103
44	Succinic acid	2.39 ppm (s, 4 H)	110-15-6	YMDB00338	HMDB00254	C00042
45	Trehalose	3.44 ppm (t, J=9.3 Hz, 2 H); 3.59 - 3.92 ppm (mm, 10 H); 5.18 ppm (d, J=3.8 Hz, 2 H)	99-20-7	YMDB00008	HMDB00975	C01083
46	Uracil	5.79 ppm (d, J=7.8 Hz, 1 H); 7.53 ppm (d, J=7.7 Hz, 1 H)	66-22-8	YMDB00098	HMDB00300	C00106
47	Ureidosuccinic acid	2.42 pm (d, J=9.7 Hz, 0.44 H); 2.46 ppm (d, J=9.7 Hz, 0.56 H); 2.64 ppm (J=3.9 Hz, 0.57 H); 2.68 ppm (d, J=4.0 Hz, 0.43 H)	13184-27-5	YMDB00027	HMDB00828	C00438
48	Uridine	5.87-5.92 ppm (mm, 2H); 7.87 ppm (d. J=8.5 Hz, 1 H)	58-96-8	YMDB00127	HMDB00296	C00299
49	Unknown-1	8.37 ppm (s, 1 H)	-	-	-	-
50	Unknown-2	8.03 ppm (s, 1 H)	-	-	-	-

*Code for acyl-carnitine is from Acyl(C12:0)-carnitine

**Code for fatty acid is from C12:0

mm = modelled multiplete

ms = multiplete modelled as deconvoluted singuletes

nd = assignment from TOCSY/HMBC/HSQC spectra (too overlapped in the ^{1}H NMR spectrum).

Note: Spectroscopic constants relative to strong coupled protons are described as various set of regular coupled proton (ex: a *dd* is expressed as two *d* with different proton integrals).

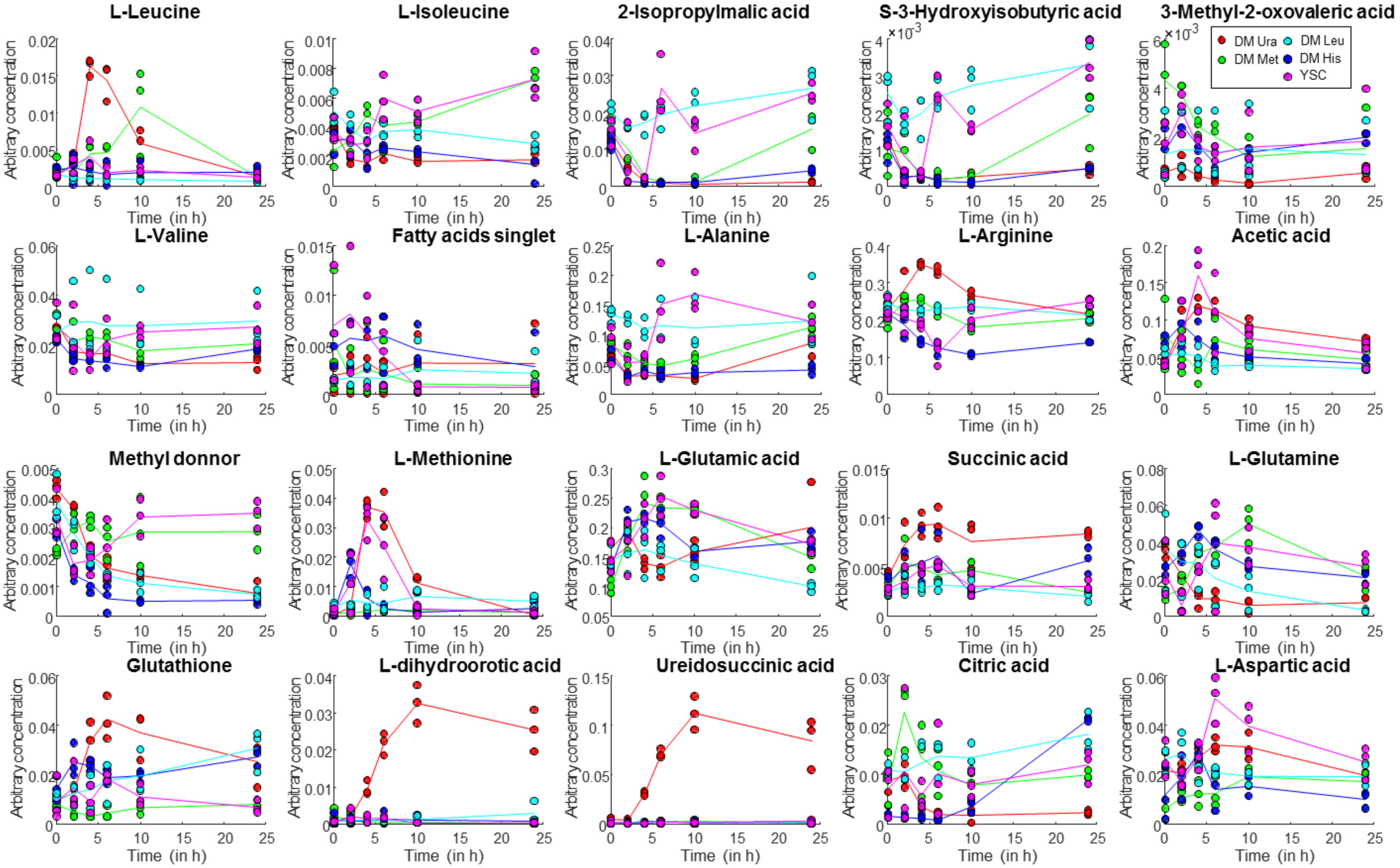

Figure S3A. Concentration estimates over time of 20 assigned metabolites.

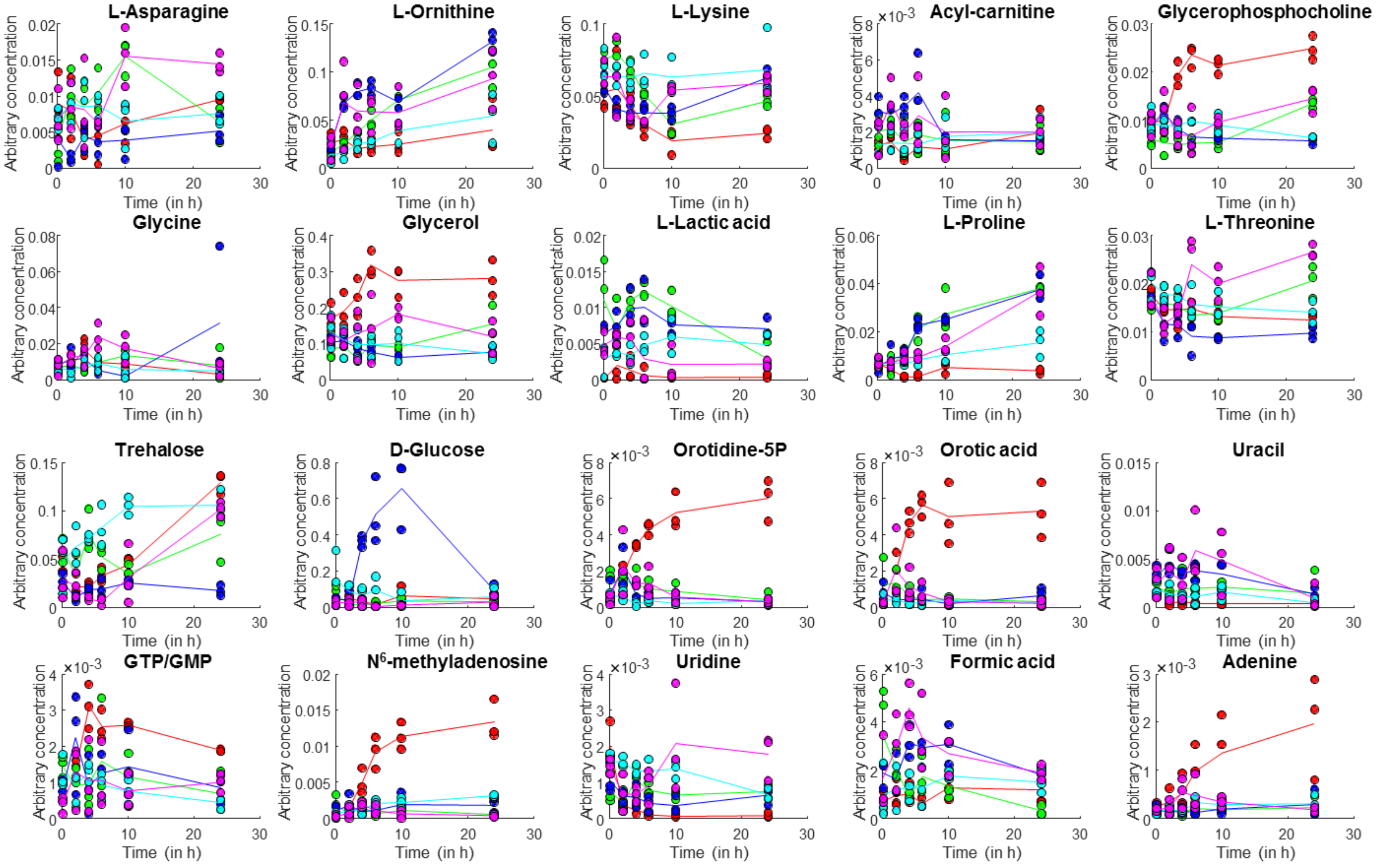

Figure S3B. Concentration estimates over time of 20 assigned metabolites. Continuation from **Fig S2A.**

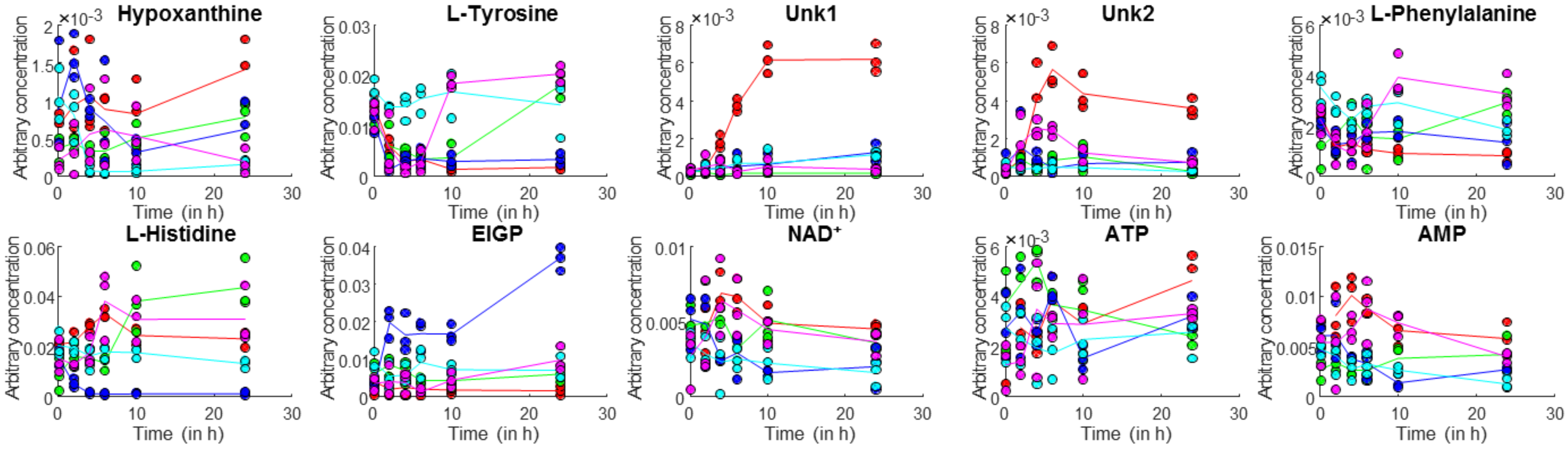

Figure S3C. Concentration estimates over time of 10 assigned metabolites. Continuation from **Fig S2B.**

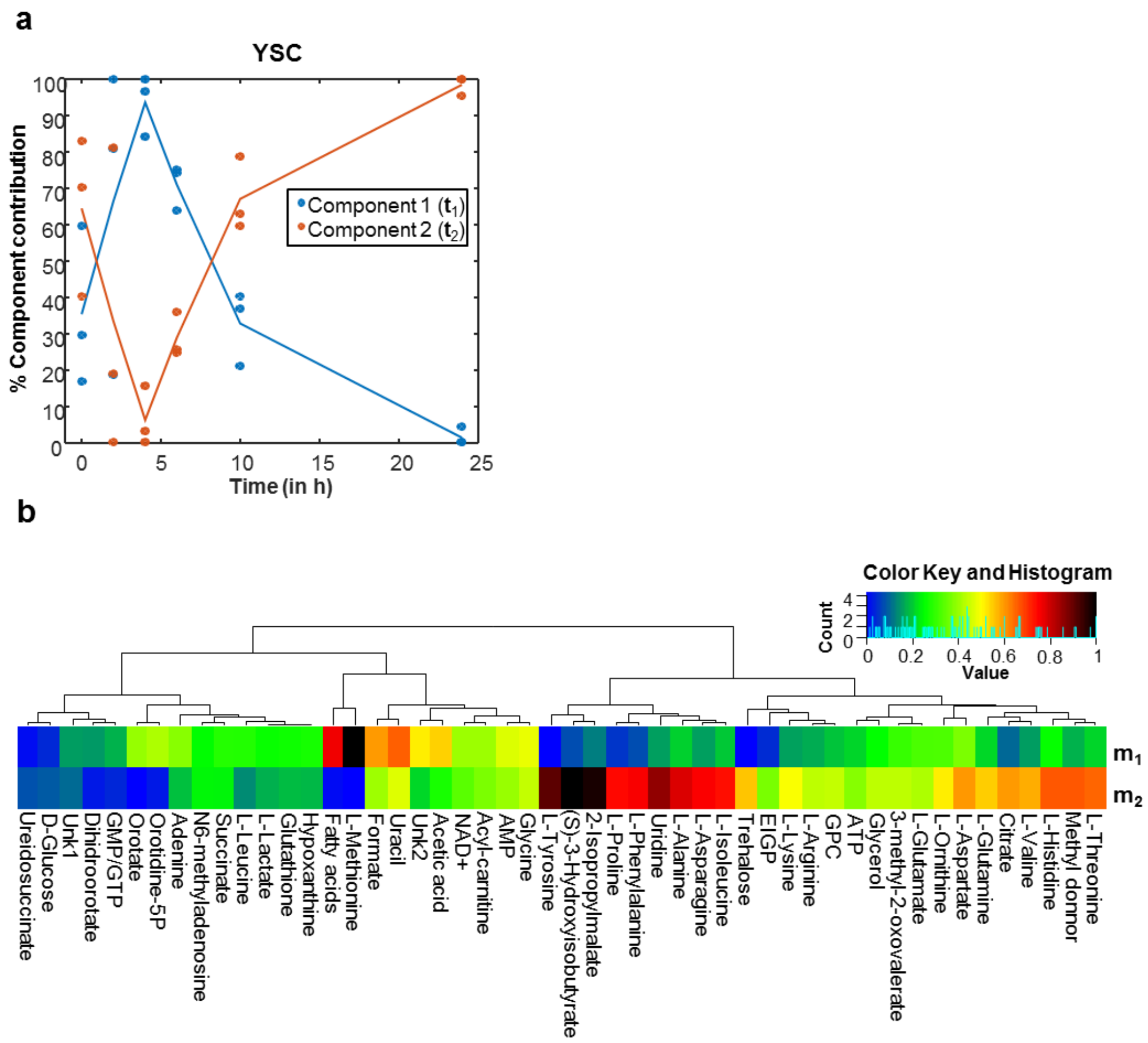

Figure S4. Growth pattern of yeast metabolism cultured in YSC using 2 components.

(**a**) Temporal growth pattern (in %). (**b**) Hierarchical clustering of the relative contribution of every metabolite in the MCR-ALS resolved components from X_{YSC} data matrix.
